# Philosophical reflections on therapeutic brain stimulation

**DOI:** 10.3389/fncom.2014.00054

**Published:** 2014-05-14

**Authors:** Walter Glannon

**Affiliations:** Department of Philosophy/Arts, University of CalgaryCalgary, AB, Canada

**Keywords:** agency, behavior control, circuit disorders, identity, neurological disorders, neurostimulation, psychiatric disorders

Neurostimulation techniques have contributed to neural models of motor, cognitive, affective and volitional functions by elucidating their biophysical underpinning. Deep brain stimulation (DBS) is particularly significant in this regard because it can both probe and modulate activity in dysfunctional neural circuits in patients with neurological and psychiatric disorders (Lozano and Lipsman, 2013) These are increasingly being understood as disorders caused by overactivity or underactivity in critical nodes of these circuits. DBS can restore circuit integrity in motor disorders such as Parkinson's disease and mood disorders such as major depression when they have been resistant to pharmacological treatment. Unilateral or bilateral stimulation of the subthalamic nucleus and globus pallidus interna to modulate dysfunction in the basal ganglia has resulted in significant improvement in motor function for many patients with Parkinson's and other movement disorders. Although it is still experimental for treating psychiatric disorders, stimulation modulating overactive regions of the prefrontal cortex and underactive regions of the subcortical reward system has resulted in improved mood and relief of anhedonia and avolition in some patients with depression.

Yet by altering functions in the neural circuits that mediate mental and motor capacities, DBS challenges the philosophical conviction that we have conscious control of our behavior. In addition, by altering the properties associated with mood and motivation, DBS may undermine the unity and continuity of the psychological properties that make each of us a unique person. Even when the therapeutic benefits of stimulating the brain outweigh the risks of adverse neurological and psychological effects, it raises metaphysical and ethical questions about agency and identity.

Many philosophers argue that agency presupposes that our actions are not generated by causal routes that bypass conscious control of the mental states that issue in them (Mele, 1995, 2009; Fischer and Ravizza, 1998, p. 236). Presumably, this would include manipulation of the brain by an artificial implanted device (Klaming and Haselager, 2013). The fact that a device can regulate a wide range of functions might suggest that it is not the person but the device that controls the person's thought and behavior. Because of its role in restoring and maintaining circuit integrity, the stimulator seems to introduce a “third party” that comes between what the patient believes is a direct connection between their brain and mind (Lipsman and Glannon, 2013). This appears to diminish if not exclude the causal role of one's conscious mental states in one's actions. Yet neurostimulation does not threaten but can restore the relevant control when it has been lost or impaired from a neurological or psychiatric disorder. By ensuring that the neural circuits mediating motor, cognitive, affective and volitional functions are neither overactive nor underactive, the modulating effects of DBS can release constraints on the ability to initiate and execute action plans. Insofar as the stimulation generates mental states that one endorses as the genuine springs of one's actions, it ensures that the actions are autonomous and that one can be responsible for them. Moreover, insofar as the stimulating system integrates with circuits regulating somatosensory and proprioceptive feedback from the body to the brain, it can restore the phenomenology of agency, the feeling of being in control of one's actions.

In DBS for Parkinson's, the fact that modulation of neural circuits operates outside of conscious awareness does not undermine control of motor functions. This is because the implicit knowledge that the electrodes are implanted and activated in the brain does not figure in the explicit content of the patient's awareness when unreflectively and automatically performing bodily movements. One need not be aware of being in control to actually be in control of one's actions. Similarly, individuals with major depression or obsessive-compulsive disorder are not aware of the electrodes modulating neural circuits and associated cognitive and affective processes. Complementary conscious and unconscious processes regulated by re-entrant loops in cortical and subcortical networks are necessary to control thought and behavior (Spence, 2009). Obsessive-compulsive disorder is a good illustration of this point. Individuals with this disorder are inhibited in performing actions they used to perform as a matter of course due to hyper-reflective thought caused by dysfunction in frontal-striatal pathways mediating cognitive and motor functions (Fuchs, 2011). DBS can re-establish normal activity in these pathways and release individuals from the paralyzing obsessions and compulsions that characterize this disorder. Theoretically, it does not matter whether mental and motor functions are generated and sustained by a natural or artificial system. Provided that an artificial system such as DBS connects in the right way with the neural inputs and outputs that regulate behavior, it can ensure that one is the agent and in control of the relevant functions.

Nevertheless, overstimulating circuits at higher frequencies, or stimulating the wrong circuits, can cause a different pathology with equally disabling effects on agency. Some individuals receiving DBS for Parkinson's disease have regained motor control but have developed compulsive and addictive behavior because nodes of the reward system were inadvertently overstimulated (Muller and Christen, 2011; Castrioto et al., 2014). A similar effect has occurred in some patients with depression, where overstimulation of the reward system has resulted in hypomania and mania (Christen et al., 2012; Synofzik et al., 2012). Neural targets must be carefully selected and stimulation parameters adjusted in response to brain changes and neurological and psychological symptoms to maintain optimal levels of neural and mental functions. Stimulation must sustain the neural and psychological mean between extremes.

The concern about DBS altering the psychological properties constitutive of diachronic personal identity is particularly germane to psychiatric disorders. The effects of stimulation can disrupt the unity and continuity of the properties in virtue of which one experiences oneself persisting through time as the same individual (Parfit, 1984). When one has experienced depression for years, one may gradually come to identify with the defining symptoms of the disorder as forming the core of one's self. In most cases, though, patients want to rid themselves of these symptoms and reclaim the phenomenology and content of the mental states they had before the onset of the disorder. It is not stimulation but the disorder that disrupts connections between the psychological properties constituting one's self by generating pathological states of mind. Stimulation can cause salutary changes in thought and behavior congruent with what one desires and would endorse as one's own. DBS can thus re-establish the person's identity (Witt et al., 2013). Indeed, the stimulating system itself becomes part of one's identity because the patient can perceive it as an enabling device that becomes integrated into his or her brain and mind (Lipsman and Glannon, 2013). Instead of an alien object, it can be described as a form of extended or expanded embodiment. The poor mood, anxiety and lack of motivation characteristic of many psychiatric disorders are ego-dystonic in the sense that there is incongruity between one's actual states and those one wants to have. Individuals with these disorders may feel that their body and mind have been hijacked by a dysfunctional brain. The insight associated with this experience can motivate them to seek therapy in the form of DBS, whose modulating effects can restore the psychological properties of their pre-morbid selves. Adverse effects of stimulation such as mania or suicidal ideation would preclude insight into the disorder and be equally disruptive of identity because they would be equally incongruent with the properties a healthy individual would persistently endorse over time. This again underscores the need for careful use of neurostimulation in targeting the right circuits with the right frequency to produce optimal levels of mental and physical functions.

To be sure, the often rapid and substantial changes in personality and behavior from DBS can make for a difficult period of adjustment for both patients and caregivers who have become accustomed to a chronic pattern of symptoms. Still, provided that the changes are beneficial and improve their quality of life, it clearly would be preferable to have to adjust to what is essentially a restoration of the patient's real self than to continue dealing with the challenges of living with a mentally and physically disabling pathology. Technological advances, such as closed loop devices that can recalibrate in response to changes in the brain while circuits are being stimulated, will reduce the incidence of adverse effects and be more tailored to individualized therapy in maximizing benefit and minimizing harm. By modulating dysfunctional neural circuits associated with neurological and psychiatric disorders, DBS can restore the motor and mental functions necessary for autonomous agency and the mental states with which one would want to identify.

The ability of DBS to probe and modulate neural circuits and alter the mental states associated with agency and identity has a deeper metaphysical implication. The fact that these states have a neural underpinning casts doubt on the idea of an immaterial human soul. Alteration of higher conscious perception by a millivolt of electrical current confirms the material nature of our consciousness (Churchland, 2013). DBS not only validates that neurological and psychiatric disorders are disorders of neural circuitry but also that the very nature and content of our mental states depends on the function or dysfunction of these circuits.

## Conflict of interest statement

The author declares that the research was conducted in the absence of any commercial or financial relationships that could be construed as a potential conflict of interest.
